# Evidence of Nrf2/Keap1 Signaling Regulation by Mitochodria-Generated Reactive Oxygen Species in RGK1 Cells

**DOI:** 10.3390/biom13030445

**Published:** 2023-02-27

**Authors:** Hiroko P. Indo, Daisuke Masuda, Sompong Sriburee, Hiromu Ito, Ikuo Nakanishi, Ken-ichiro Matsumoto, Samlee Mankhetkorn, Moragot Chatatikun, Sirirat Surinkaew, Lunla Udomwech, Fumitaka Kawakami, Takafumi Ichikawa, Hirofumi Matsui, Jitbanjong Tangpong, Hideyuki J. Majima

**Affiliations:** 1 Department of Oncology, Graduate School of Medical and Dental Sciences, Kagoshima University, Kagoshima 890-8544, Japan; 2 Amanogawa Galactic Astronomy Research Center (AGARC), Graduate School of Sciences, Kagoshima University, Kagoshima 890-0065, Japan; 3 Department of Space Environmental Medicine, Graduate School of Medical and Dental Sciences, Kagoshima University, Kagoshima 890-8544, Japan; 4 Utilization & Engineer Department, Japan Manned Space Systems Corporation, 21-6 Tsukuba, Tsukuba 305-0047, Japan; 5 Department of Radiologic Technology, Faculty of Associated Medical Sciences, Chiang Mai University, Chiang Mai 50200, Thailand; 6 Quantum RedOx Chemistry Team, Institute for Quantum Life Science, Quantum Life and Medical Science Directorate, National Institutes for Quantum Science and Technology, Chiba 263-8555, Japan; 7 Quantitative RedOx Sensing Group, Department of Radiation Regulatory Science Research, National Institute of Radiological Sciences, Quantum Life and Medical Science Directorate, National Institutes for Quantum Science and Technology (QST), Chiba 263-8555, Japan; 8 School of Allied Health Sciences, Walailak University, Thasala, Nakhon Si Thammarat 80160, Thailand; 9 Center of Excellence Research for Melioidosis and Microorganisms, Walailak University, Thasala, Nakhon Si Thammarat 80160, Thailand; 10 Research Excellence Center for Innovation and Health Products (RECIHP), School of Allied Health Sciences, Walailak University, Thasala, Nakhon Si Thammarat 80160, Thailand; 11 School of Medicine, Walailak University, Thasala, Nakhon Si Thammarat 80161, Thailand; 12 Department of Regulation Biochemistry, Graduate School of Medical Sciences, Kitasato University, 1-15-1 Kitasato, Sagamihara 252-0373, Japan; 13 Department of Health Administration, School of Allied Health Sciences, Kitasato University, 1-15-1 Kitasato, Sagamihara 252-0373, Japan; 14 Regenerative Medicine and Cell Design Research Facility, School of Allied Health Sciences, Kitasato University, 1-15-1 Kitasato, Sagamihara 252-0373, Japan; 15 Division of Gastroenterology, Graduate School of Comprehensive Human Sciences, University Tsukuba, Tsukuba 305-8575, Japan

**Keywords:** mitochondria, reactive oxygen species, tumorized cells, gastric mucosal cells, cell signaling, signal transduction, Nrf2/Keap1

## Abstract

It has been known that reactive oxygen species (ROS) are generated from the mitochondrial electron transport chain (ETC). Majima et al. proved that mitochondrial ROS (mtROS) caused apoptosis for the first time in 1998 (Majima et al. J Biol Chem, 1998). It is speculated that mtROS can move out of the mitochondria and initiate cellular signals in the nucleus. This paper aims to prove this phenomenon by assessing the change in the amount of manganese superoxide dismutase (MnSOD) by MnSOD transfection. Two cell lines of the same genetic background, of which generation of mtROS are different, i.e., the mtROS are more produced in RGK1, than in that of RGM1, were compared to analyze the cellular signals. The results of immunocytochemistry staining showed increase of Nrf2, Keap1, HO-1 and 2, MnSOD, GCL, GST, NQO1, GATA1, GATA3, GATA4, and GATA5 in RGK1 compared to those in RGM1. Transfection of human MnSOD in RGK1 cells showed a decrease of those signal proteins, suggesting mtROS play a role in cellular signals in nucleus.

## 1. Introduction

Oxidative stress contributes to several acute and chronic diseases, including cancer, alcoholic liver disease, Crohn’s disease, rheumatoid arthritis, diabetes, muscular dystrophy, cystic fibrosis, septic shock, premature birth, atherosclerosis, infertility, cataracts, aging, hepatitis, acute respiratory distress syndrome, ischemia, and neuronal degeneration in Parkinson’s and Alzheimer’s disease [1]. The concept of oxidative stress dates back to Sies [2], and oxidative stress and the principles of protection against it have been discussed for normal cells and tumors [1]. In most cancer cells, intracellular energy production is predominantly achieved by glycolysis, followed by lactic acid fermentation in the cytosol, even under aerobic conditions through a phenomenon known as the “Warburg effect” [3,4]. Thus, it appears that the oxidative phosphorylation process is defective in cancer cells. A previous study revealed an increase in the production of reactive oxygen species (ROS) in the mitochondria with an impaired electron transport chain (ETC) and mitochondrial DNA damage [5]. In normal cells, and even cancer cells, oxidative stress is severe, and ROS levels from the mitochondria are expected to increase.

**Figure 1:**
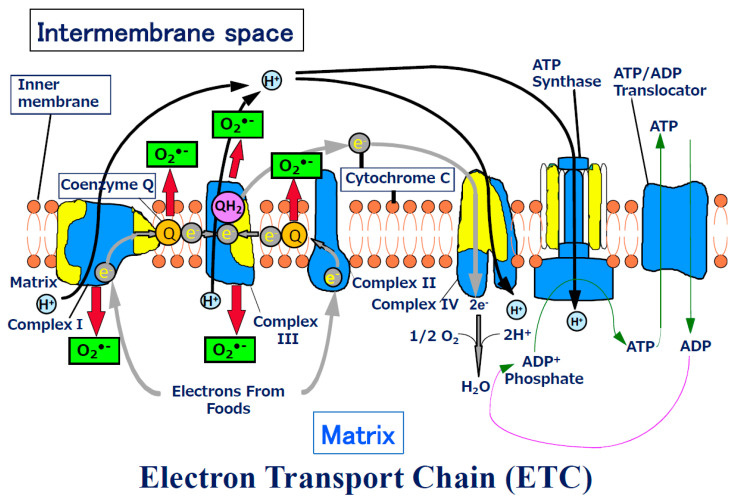
Schema of Electron Transport Chain. ATP production by oxidative phosphorylation with an electron transport chain (ETC). The ETC is composed of over 90 subunits. Among the subunits, yellow parts are the subunits those are translated by the genes in mitochondrial DNA. NADH and FADH2 carry protons (H^+^) and electrons (e^−^) to the electron transport chain located in the inner membrane. The energy from the transfer of electrons along the chain transports the protons across the membrane and creates an electrochemical gradient. As the accumulating protons follow the electrochemical gradient back across the membrane through an ATP synthase complex, their movement provides energy for synthesizing ATP from ADP and phosphate. At the end of the electron transport chain, two protons, two electrons, and half of an oxygen molecule combine to form water. Then 2–3% of electrons leak from ETC, and oxygen traps the electrons and become superoxide (O_2_^•−^ ). O_2_^•−^ can change its form to become various reactive oxygen species (ROS) in mitochondria.

The ETC is composed of over 90 subunits [6]. The emergence of the mitochondrion resulted in increased production of ATP by the ETC in the mitochondrial inner membrane in the presence of oxygen [7]. In the glycolytic pathway, only two ATP molecules are produced, while ETC produces 36; thus, a total of 38 molecules of ATP are produced. The ETC consists of complexes I–IV, ATP synthase, and ANP translocator (Figure 1). The mitochondrion also has DNA, which encodes 13 of these proteins. The mtDNA contains codon sequences different from those of the nuclear DNA. In the mitochondrial ETC, while oxidation/reduction in electrons occurs repeatedly, hydrogen ions are transported into the intermembrane space and finally back to the matrix, and ADP is converted into ATP using the produced energy. The produced ATP is transported into the intermembrane space (Figure 1). This complicated ‘bio-machine’ serves as the largest energy-producing organelle of the cell but is an imperfect machine and thus causes a ‘leakage’ of electrons. This leakage most frequently occurs in complexes I and III. The 2–3% of electrons leak even in a normal state, and superoxide is considered to be generated from these leaked electrons [1,8,9,10,11]. Oxygen traps the electrons, thus, becoming a superoxide anion (O_2_^•−^). It is known that Complex I generates O_2_^•−^ in the matrix [12], and Complex III generates O_2_^•−^ into both the matrix and inner membrane space [13]. Coenzyme Q works as both antioxidant [14] and a pro-oxidant [15], and it is known that it produces superoxide [16]. Further, Linnane et al. suggested that coenzyme Q produces superoxide, probably in the intermembrane space, and commits to the transcription of genes and gene regulations [14].

It is known that in chemotherapy, once cancer cells have been exposed to a drug, they become resistant to further treatment [17]. Similarly, after cells are dosed with radiation, they become resistant to further doses [18,19]. These phenomena may explain cells responding to severe oxidative stress and must repeatedly respond subsequently to a series of oxidative insults. We focus on mitochondria to play these roles. Previously we reported that oxidative stress is capable of causing cell death. Majima et al. [20] reported the first evidence of a relationship between mitochondrial ROS (mtROS) and apoptosis. Manganese superoxide dismutase (MnSOD) scavenges ETC-generated superoxide molecules. Cells transfected with MnSOD exhibit increased resistance to paraquat [21], tumor necrosis factor [22,23], doxorubicin [23], mitomycin C [23], irradiation [23,24,25,26,27,28], alkaline treatment [20], ischemia–reperfusion injury [29], cigarette smoking toxicity [30], and radiation carcinogenesis [31]. Transgenic mice carrying the human MnSOD (hMnSOD) gene show a reduced severity of hyperbaric oxygen-induced pulmonary damage [32] or doxorubicin-induced myocardial damage [33]. Free radicals generated in mitochondria possibly play roles in all morphological processes of cell death, i.e., apoptosis, necrosis, and autophagy [34,35].

mtROS control cell death; however, mtROS controlling cellular signaling is under debate. ROS formation may also act as a pre-conditioner that helps prevent or ameliorate secondary or subsequent oxidative stress events. This may occur via up-or down-regulation of cell signaling pathways, and some of these processes may be driven, either directly or indirectly, via mitochondrial ROS formation and its downstream consequences. While it is known that ROS can initiate various signal transduction pathways, the role of mitochondrial ROS in initiating signal transductions in the cell cytosol has been the subject of controversy. This subject has been dealt with in several reviews [36,37,38,39,40,41,42,43]. However, all of these papers are hypothetical papers or just ideas. No authors proved that mtROS initiate the signals in the nucleus. The only way to change the amount of mtROS is to change the amount of MnSOD that again scavenges superoxide ions generated from mitochondrial ETC. MtROS can initiate the signals in the nuclei, which can be proved by MnSOD transfection and see a decrease in the signals in nuclei. Therefore, this manuscript aims to transfect MnSOD wherein mtROS production is decreased and to see if the intracellular signaling, such as nuclear factor-erythroid 2-related factor 2 (Nrf2), Kelch-like ECH-associated protein 1 (Keap1), HO-1 and 2, MnSOD, GCL, GST, NQO1, GATA1, GATA3, GATA4, and GATA5 decrease. Whether mtROS initiate cellular signals in nuclei is a fascinating subject, and we will attempt to see if the augmentation of signaling in a nucleus can be suppressed by MnSOD transfection.

We previously established a rat gastric mucosal cell line, RGM1 [44], and a tumorized cell line, RGK1, which was derived from the RGM1 cell line [45] and described how ROS activate cellular signaling and molecules [46]. RGM1 and RGK1 have the same genetic background. In this paper, RGK1 is used because the cells generate more mtROS, and not for the cancer cell line. In normal situations, the augmentation of the generation of mtROS is possible by various stimuli [5]. Focusing on the mtROS, we use RGM1 and RGK1 as models of one producing less mtROS (RGM1) and the other producing more mtROS (RGK1). In this study, using both RGM1 and RGK1 cells, we aim to examine the effects of mtROS on the activation of signal transcription in the nucleus.

## 2. Materials and Methods

### 2.1. Cell Lines and hMnSOD Transfection

RGM1 is a diploid untransformed cell line derived from normal gastric mucosa of wistar rats [44]. The RGK1 cell line established as an *N*-methyl-*N*′-nitro-*N*-nitrosoguanidine-induced mutant of RGM1, can be used as an in vitro model of gastric cancer [45]. A plasmid derived from pCR3.1 (Invitrogen, Waltham, MA, USA) and containing a sense hMnSOD cDNA insert, pCR3.1-Uni, was kindly provided by Dr. Makoto Akashi (National Institute of Radiological Sciences, Chiba, Japan) for this study. The hMnSOD sequence was identical to that in the Kyoto Encyclopedia of Genes and Genomes (locus link ID: 6648; http://www.genome.jp/dbget-bin/www_bget?hsa+6648, accessed on 30 April 2021). RGM1 and RGK1 cells were transfected using Lipofectamine (Invitrogen) according to the manufacturer’s instructions. Briefly, cells were plated 24 h before transfection at 80% confluence in a 60 mm dish. Cells were transiently transfected with 8 µg pCR3.1-Uni in serum-free Dulbecco’s modified Eagle medium and Ham’s F12 medium (DMEM/F12; Cosmo Bio, Tokyo, Japan). Controls were transfected with a pCR3.1 vector lacking the hMnSOD cDNA insert. Six hours after transfection, the medium was changed to DMEM/F12 containing 10% fetal bovine serum (JRH Biosciences, Denver, PA, USA). After 24 h, cells were treated with trypsin and plated for use in all subsequent experiments.

### 2.2. Relative Levels of Mitochondrial ROS

HPF (Daiichi Pharmaceutical Co., Tokyo, Japan) is a new fluorescent dye used for the selective detection of hydroxyl radicals that was recently developed by our group [47] and was used to that end in the present study. Hydroxyl radicals are highly reactive oxygen species (hROS). The dynamic range of fluorescence augmentation for such a dye is expected to be broad. Although HPF itself fluoresces to only a small extent, it selectively and dose-dependently yields a strongly fluorescent compound, fluorescein, on reaction with hROS, but not other ROS. Glass-bottomed (35 mm) dishes (MatTek Corp., Ashland, MA, USA) with cell monolayers were prepared for staining with HPF. Twenty-four hours after plating the cells, the cell culture medium was replaced with a modified Hanks’ balanced salt solution containing 10.0 mM HEPES, 1.0 mM MgCl_2_, 2.0 mM CaCl_2_, and 8.3 mM glucose, adjusted to pH 7.30 ± 0.05. HPF (10 µM) was added to the cells and incubated for 15 min at 37 °C. Bioimages were obtained using a CSU-10 confocal laser scanning unit (Yokogawa Electric Co., Tokyo, Japan) coupled to an IX90 inverted microscope with a 20x UPlanApo objective lens (Olympus Optical Co., Tokyo, Japan) and a C5810-01 color chilled 3CCD camera (Hamamatsu Photonics, Hamamatsu, Japan). HPF was excited at 488 nm, and emissions were filtered using a 515 nm barrier filter. The intensity of the laser beam, the exposure time of the 3CCD camera, and the gain of the amplifier were set at 500 µW, 1.0 s, and 18 decibels, respectively, to allow quantitative comparisons of the relative fluorescence intensity of the cells between groups. Cells were chosen on a random basis, and a total of over 250 cells were analyzed to detect fluorescence values. Average fluorescence intensity per cell was determined using IPLab Spectrum version 3.0 software (Scanalystics Inc., Fairfax, VA, USA) with some program modification by one of the authors (H.J.M).

### 2.3. Immunofluorescence Staining

Glass-bottomed (35 mm) dishes (MatTek Corp) with cell monolayers were prepared for immunofluorescent staining. The cells were fixed with 4% formaldehyde saline (PBS) solution at 25 °C for 30 min and rinsed twice with PBS; membranes were permeabilized using 95% ethanol with 5% acetic acid for 10 min. After washing twice with PBS, cells were incubated for 30 min in a blocking serum solution (1% bovine serum albumin in PBS), then for 1 h at room temperature with anti-Nrf2 (rabit polyclonal IgG, SC-13032, Santa Cruz Biotechnology, Dallas, TX, USA), anti-Keap1 (goat polyclonal IgG, SC-15246, Santa Cruz), anti-heme oxygenase (HO)-1 (rabit polyclonal IgG, cat#HC3001, BIOMOL International, Plymouth Meeting, PA, USA), anti-HO-2 (rabit polyclonal IgG, cat#HC3002, BIOMOL), anti-NAD(P)H quinone dehydrogenase 1 (NQO1) (mouse IgG, cat#611426, Novus Biologicals, Englewood, CO, USA), anti-glutamate-cysteine ligase (GCL) (NeoMarkers, Fremont, CA, USA), anti-glutathione S-transferase (GST) (mouse monoclonal IgG, cat#HC3001, Cell Signaling Technology, Danvers, MA, USA), anti-MnSOD (mouse polyclonal IgG, cat #06-984, Millipore, Burlington, MA), anti-GATA1 (mouse monoclonal IgG, SC-266, Santa Cruz Biotechnology, Dallas, TX, USA) [40], anti-GATA3 (mouse monoclonal IgG, SC-268, Santa Cruz Biotechnology, Dallas, TX) [40], anti-GATA4 (goat polyclonal IgG, SC-1237, Santa Cruz Biotechnology, Dallas, TX, USA) [40], and GATA5 (goat polyclonal IgG, SC-7280, Santa Cruz Biotechnology, Dallas, TX, USA) [40], each at a dilution of 1:200. Cells were rinsed twice with 1% bovine serum albumin in PBS and then incubated as appropriate with Alexa-Fluor-488-labeled goat anti-mouse IgG (H+L), Alexa-Fluor-488-labeled donkey anti-goat IgG (heavy and light chains; H+L) or Alexa-Fluor-488-labeled goat anti-rabbit IgG (H+L) (Molecular Probes, Eugene, OR, USA) for 1 h at 25 °C in the dark. Image acquisition and analysis were performed as for HPF, except that the exposure time was 4 s.

### 2.4. SOD Activity Gel Assay

A native gel-based assay for examining SOD activity was carried out according to a previously described method [48] with slight modifications. Cells were sonicated in a 50 mM potassium phosphate buffer (pH 7.8). Cell protein (20 µg/lane) was run on a native riboflavin gel comprising a 5% stacking gel (pH 6.8) and a 12% running gel (pH 8.8) at 4 °C. To visualize SOD activity, gels were first soaked in 2.43 mM nitro blue tetrazolium (Wako Pure Chemical Industries, Osaka, Japan) in deionized water for 20 min and then in 56 nM riboflavin (Wako Pure Chemical Industries, Ltd., Osaka, Japan) with 28 mM *N*,*N*,*N*′,*N*′-tetramethylethylenediamine (Sigma Aldrich, St. Louis, MO, USA) in a 50 mM potassium phosphate buffer (pH 7.8) for 15 min in the dark. Gels were washed with deionized water and illuminated under fluorescent light until clear zones of SOD activity were evident. The images were recorded and MnSOD bands were quantified using the AlphaImager imaging system (Alpha Innotech, San Leandro, CA, USA). MnSOD activity was assessed by examining the band density. The MnSOD activity of vector-only-transfected control cells was normalized to a value of 1.0 and relative MnSOD activities in other cells were calculated. Results were calculated as the mean of the integrated density from five independent runs.

### 2.5. Isolation of the Total RNA

Total RNA was isolated using ISOGEN from cultured cells as recommended by the manufacturer (Nippon gene, Toyama, Japan).

After washing cells by PBS 3 times, 1 mL of ISOGEN was added to cells and collected into 1.5 mL tubes. Two hundred μL of chloroform was added to the samples and vortexed. After centrifugation at 12,000× *g* for 15 min at 4 °C, the aqueous phase was transferred to new 1.5 mL tubes and 0.5 mL of 2-propanol was added. After incubation for 5 min at 25 °C, centrifugation was performed at 12,000× *g* for 10 min at 4 °C to precipitate RNA. The pellets were washed with 70% of EtOH and after centrifugation 7500× *g* for 5 min at 4 °C, pellets were dried briefly and dissolved in TE. The RNA quality was checked by measuring 260 nm absorbance and electrophoresis. All cDNAs were prepared by reverse transcription of 1 μg total RNA using oligo dT(20) primer (0.4 μM/50 μL final volume), and ReverTra Ace (TOYOBO) as recommended by the manufacturer. An equivalent volume of 0.1 μL of cDNA solution was used for quantification of specific cDNA by qRT-PCR.

### 2.6. qRT PCR for hMnSOD and rMnSOD (Control) mRNA Detection

Total RNA was isolated from cells, and cDNA was synthesized as described above. The sequences of primers were compared to those from the available human genome database (https://www.genome.jp/kegg/genes.html, assessed on 31 January 2020) in order to select primers that would produce a single amplification product. The forward and reverse primers are in Table 1.

The qRT-PCR assays were performed on an ABI prism 7000 Sequence Detection System (Applied Biosystems, Foster City, CA, USA) using the QuantiTect SYBR Green PCR Kit (Qiagen, Valencia, CA, USA). The experimental conditions were those recommended by the manufacturer. The resulting cDNA was amplified at the following conditions. Human MnSOD: annealing and elongation at 60 °C for 1 min, followed by 15 s at 95 °C, and 1 min at 60 °C. For rat MnSOD: annealing at 95 °C for 15 s, and elongation at 60 °C for 10 s, followed by 72 °C for 30 s. All PCR assays were performed for 40 cycles. The size of single amplification products was further verified by gel electrophoresis. All data were normalized to an internal standard (glyceraldehyde-3-phosphate dehydrogenase; GAPDH). The triplicate samples were used in an assay of qRT-PCR and repeated three times. The average values were calculated, and the bar was expressed as S.D.

### 2.7. Construction of Antioxidant Response Element (ARE) Reporter Vector

A sequence-encoding ARE was inserted at XhoI and HindIII sites of the pGL4.28[luc2CP/minP/Hygro] vector (Promega, Madison, WI, USA). The inserted sequences were

ARE-GST-Ya-F
(5′-TCGAGTAGCTTGGAAATGACATTGCTAATGGTGACAAAGCAACTTTA-3′; underlining marks the ARE consensus sequence) and
ARE-GST-Ya-R
(5′-AGCTTAAAGTTGCTTTGTCACCATTAGCAATGTCATTTCCAAGCTAC-3′) [49].

Inserts were annealed in a 1× annealing buffer (30 mM HEPES-KOH, 100 mM potassium acetate, 10 mM magnesium acetate) at 30 °C for 1 h after 90 °C for 1 min. The annealed ARE insert, and pGL4.28[luc2CP/minP/Hygro] vector digested with XhoI and HindIII were ligated using a DNA Ligation Kit, Mighty Mix (TaKaRa, Shiga, Japan). The plasmid was transformed into competent cells, and positive clones were picked. Plasmid for transfection was extracted using a QIAfilter Plasmid Midi Kit (QIAGEN, Valencia, CA, USA).

### 2.8. Dual-Luciferase Reporter Assay

Reporter assays were performed using a Dual Luciferase Reporter Assay System (Promega) and a Fluoroskan Acent FL microplate reader (Thermo Fisher Scientific, Waltham, MA, USA) according to the manufacturer’s protocol. Cultured cells were lysed in a Passive Lysis Buffer (PLB) after two washes with PBS. Cells in PLB (cell lysis sample) were collected using a Cell Lifter (Fisher Scientific). Cell lysis samples (20 µL) were transferred to a 96-well plate and 100 µL Luciferase Assay Reagent II (LARII) was added to each sample. Firefly luciferase activity was measured for 10 s, then STOP & GLO Reagent (100 µL) was added to the sample and Renilla luciferase activity was measured for 10 s.

### 2.9. Statistical Analysis

Statistical analysis was performed using the Student *t*-test. The results with *p*-values < 0.05 were considered to be statistically significant.

## 3. Results

### 3.1. Mitochondrial ROS Generation and MnSOD Activity in RGM1 and RGK1 Cells

**Figure 2:**
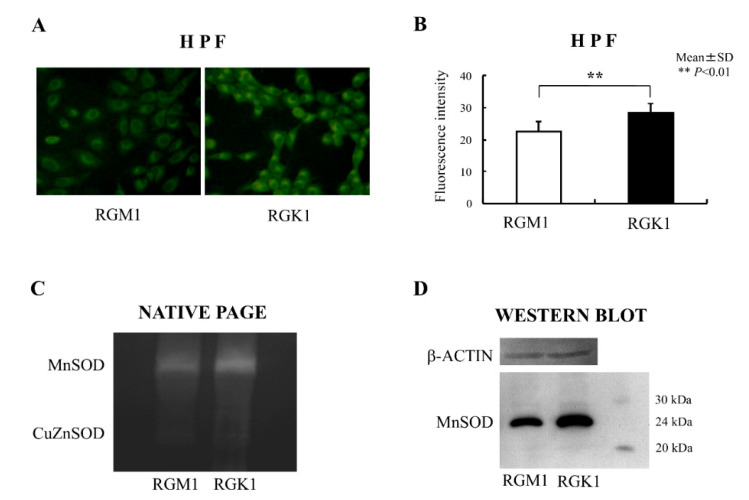
Reactive oxygen species (ROS) and MnSOD expression and activity in RGM1 and RGK1 cell lines. (**A**) Laser confocal images of cells treated with the ROS-reactive dye, HPF. HPF was loaded in RGM1 and RGK1 cells for 15 min, and the fluorescence was observed using a laser confocal microscope. Magnification 20×. (**B**) Semiquantitative fluorescent detection of ROS in the presence of HPF. The fluorescence intensity was significantly higher in RGK1 cells than in RGM1 cells. Bar: mean ± S.D.; *t*-test. **: *p* < 0.01. (**C**) SOD activity in RGM1 and RGK1 cells was analyzed by staining a native polyacrylamide gel. Each lane was loaded with 20 μg of protein and electrophoresed through the native polyacrylamide gel at 4 °C. Both activities in both cell lines were clearly detectable. The MnSOD activity of RGK1 cells was greater than that of RGM1 cells activity. (**D**) Expression of MnSOD protein was detected using Western blot analysis. Each lane was loaded with 20 μg of protein and electrophoresed on a 12% polyacrylamide gel at 4 °C. The protein levels of RGK1 cells increased compared with those in RGM1 cells (Redrawn from [50] with permission).

A dye sensitive to hydroxyl radical, HPF, was used to detect mitochondrial ROS. The dye was loaded for 15 min at 37 °C. and the images were acquired. The results showed that the fluorescence intensity of HPF was higher in RGK1 cells than in RGM1 cells [50] (Figure 2A,B). MnSOD is an enzyme essential for scavenging superoxide in mitochondria. The MnSOD protein was evaluated using a gel-based activity assay and Western blotting. MnSOD activity in RGK1 cells was higher than in RGM1 cells (Figure 2C), in parallel with higher expression of MnSOD protein, as indicated by Western blotting results (Figure 2D). The results confirm that mitochondrial ROS levels are higher in tumor cells.

### 3.2. Expression of Nrf2, Keap1, and Oxidative Stress-Related Proteins in RGM1 and RGK1 Cells

**Figure 3:**
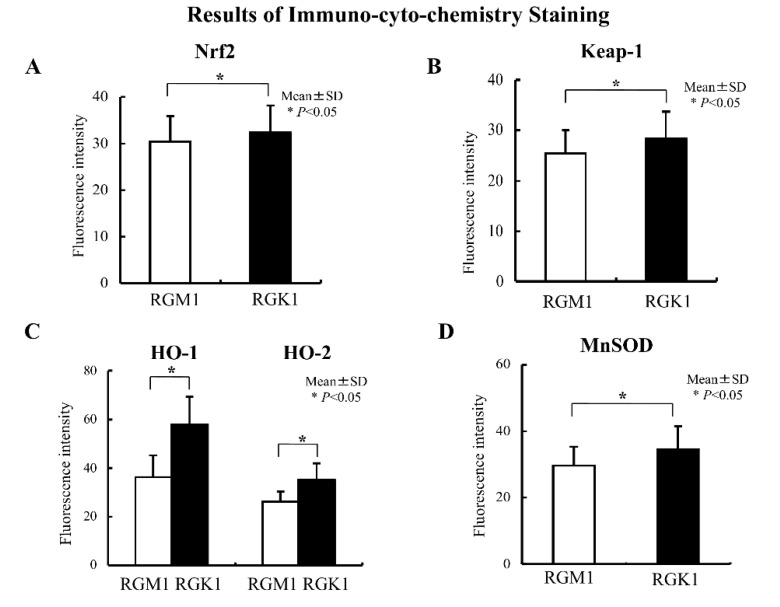
Results of immunocytochemistry staining. Immunohistochemical staining with an anti-Nrf2 antibody in RGM1 and RGK1 cell lines was performed. (**A**) The fluorescence intensity was significantly higher in RGK1 cells compared with that in RGM1 cells. Bar: mean ± S.D.; *t*-test. * *p* < 0.05. (**B**) Immunohistochemical staining with an anti-Keap1 antibody in RGM1 and RGK1 cell lines was performed. The fluorescence intensity was significantly higher in RGK1 cells compared with that in RGM1 cells. Bar: mean ± S.D.; *t*-test. * *p* < 0.05. (**C**) Immunohistochemical staining with anti-HO-1 and anti-HO-2 antibodies in RGM1 and RGK1 cell lines was performed. The fluorescence intensity for both HO-1 and HO-2 was significantly higher in RGK1 cells compared with that in RGM1 cells. Bar: mean ± S.D.; *t*-test. * *p* < 0.05. (**D**) Immunohistochemical staining with an anti-MnSOD antibody in RGM1 and RGK1 cell lines was performed. The fluorescence intensity was significantly higher in RGK1 cells compared with that in RGM1 cells. Bar: mean ± S.D.; *t*-test. * *p* < 0.05.

**Figure 4:**
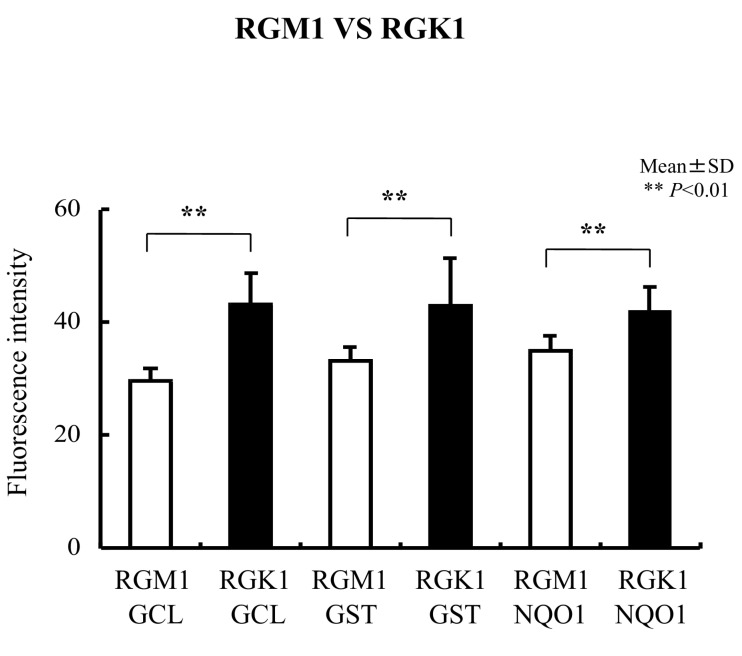
Immunohistochemical staining with anti-GCL, anti-GST, and anti-NQO1 antibodies in RGM1 and RGK1 cell lines was performed. The fluorescence intensity for GCL, GST, and NQO1 was significantly higher in RGK1 cells compared with that in RGM1 cells. Bar: mean ± S.D.; *t*-test. ** *p* < 0.01.

Levels of Nrf2 and Keap1 protein were evaluated using immunohistochemical staining. Nrf2 protein levels in RGK1 cells were significantly higher than in RGM1 cells (Figure 3A), and similar results were obtained for the Keap1 protein (Figure 3B). Comparable results were observed for the oxidative stress-related proteins HO-1 and HO-2 (Figure 3C), MnSOD (Figure 3D), NQO1, GCL, and GST (Figure 4), all of which are downstream products of Nrf2/Keap1 signaling [51].

### 3.3. MnSOD Expression and Enzyme Activity Following Transfection into RGK1 Cells

**Figure 5:**
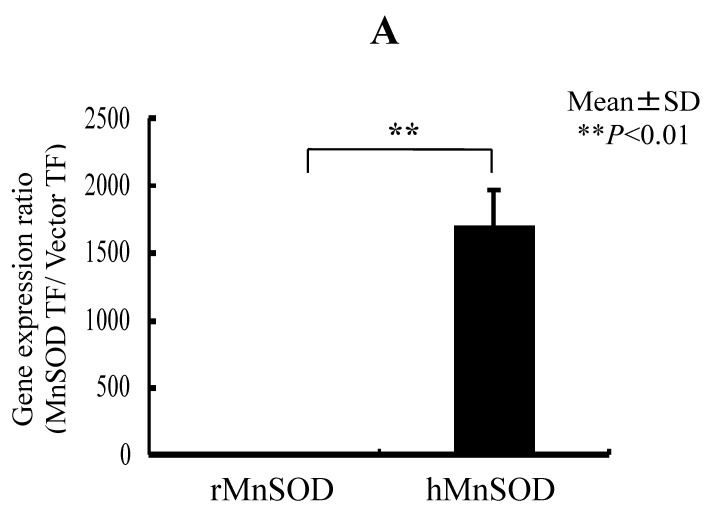

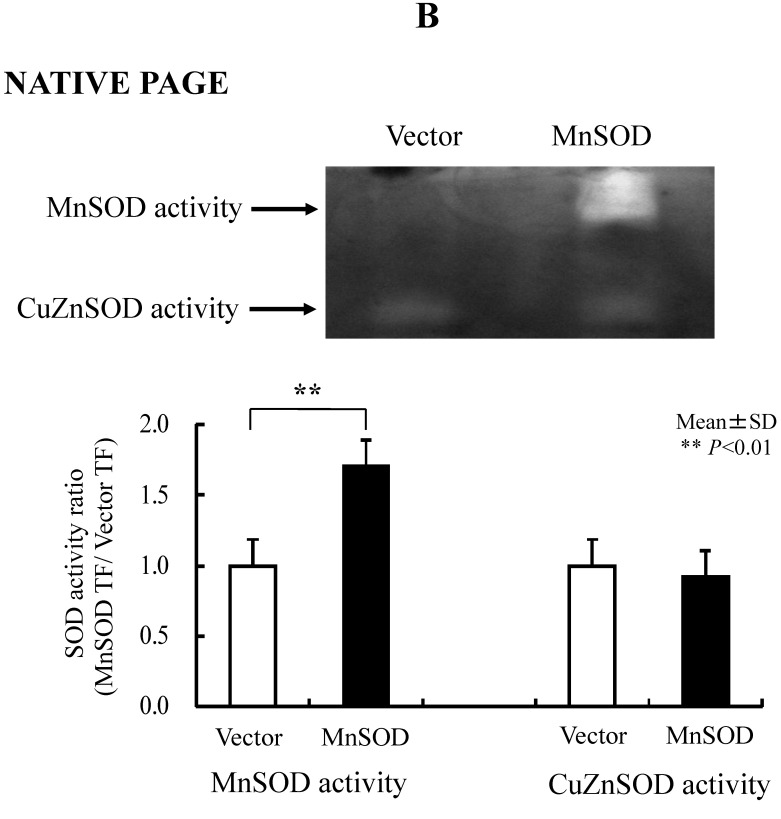
RGK1-MnSOD Gene Expression by hMnSOD cDNA Vector Transfection, and RGK1- SOD Activity by MnSOD cDNA transfection. (**A**) MnSOD expression in RGK1 cells following transfection. The gene expression ratio of hMnSOD transfectants per vector-only transfectants (MnSOD TF/Vector TF) is calculated. Bar: mean ± S.D.; *t*-test. ** *p* < 0.01. (**B**) SOD activity in RGK1 cells following transfection with vector alone or with hMnSOD vector. (Upper) Native polyacrylamide gel stained for SOD activity. (Lower) Calculated activity ratios for MnSOD and CuZnSOD. Each lane was loaded with 20 μg of protein and electrophoresed through a native PAGE blot at 4 °C. Both activities in both cell lines were clearly detectable. The MnSOD activity in RGK1 cells was higher than that in RGM1 cells. The MnSOD activity in hMnSOD-vector-transfected cells (MnSOD) was significantly higher than in cells transfected with vector alone (Vector). The CuZnSOD activity in hMnSOD-vector-transfected cells (MnnSOD) and the cells transfected with vector alone (Vector) showed no significant differences. Bar: mean ± S.D.; *t*-test. ** *p* < 0.01.

We transfected the MnSOD gene into RGK1 cells and, using decreasing ROS in mitochondria, tested whether MnSOD, by means of decreasing ROS in mitochondria, also decreases Nrf2/Keap1 signaling. The production of active MnSOD in transfected cells was investigated in cell lysates. Expression of MnSOD mRNA was measured using real-time RT-PCR analysis and expression of MnSOD protein was detected by a gel-based SOD activity assay. mRNA levels for hMnSOD in hMnSOD-transfected cells were significantly higher than in cells transfected with vector alone (Figure 5A). MnSOD activity was also significantly higher in hMnSOD-transfected cells. In contrast, the activity of copper–zinc superoxide dismutase (CuZnSOD) did not differ between cells with vector-alone and hMnSOD-transfected cells (Figure 5B).

### 3.4. Oxidative Stress-Related Protein Expression Following MnSOD Transfection into RGK1 Cells

**Figure 6:**
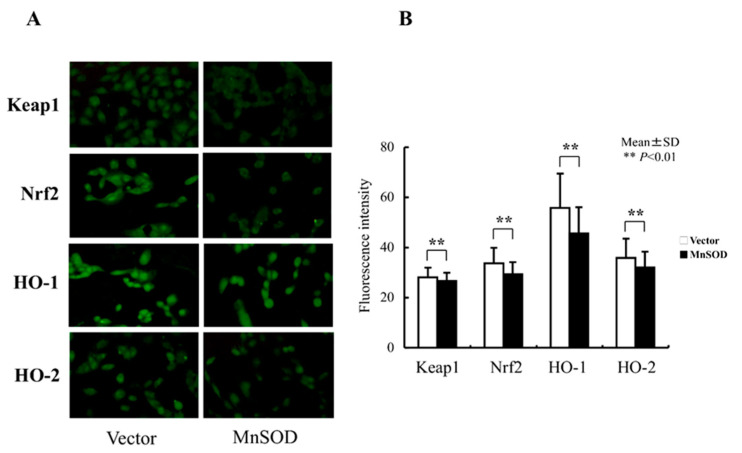
Changes in oxidative-stress-related protein expression following hMnSOD transfection into RGK1 cells. (**A**) Laser confocal images of fluorescence-stained cells. Magnification 20×. (**B**) Semiquantitative fluorescent detection of target proteins in hMnSOD-vector-transfected cells (MnSOD) and the cells transfected with vector alone (Vector). The fluorescence intensity in hMnSOD-vector-transfected cells was significantly decreased compared with that in the cells transfected with vector alone, suggesting hMnSOD gene transfection worked in mitochondria and decreased the ROS levels, resulting in lower Keap1, Nrf2, HO-1, and HO-2 fluorescence levels. Bar: mean ± S.D.; *t*-test. ** *p* < 0.01.

Levels of oxidative stress-related proteins were evaluated by immunohistochemical staining to determine whether the levels of Keap1, Nrf2, HO-1, and HO-2 proteins decreased following hMnSOD transfection into RGK1 cells. The changes in fluorescence staining in the vector-alone- or hMnSOD-vector-transfected cells, as assessed two days after transfection, indicate that expression of these proteins is suppressed in hMnSOD-vector-transfected cells (Figure 6A,B).

### 3.5. Dual-Luciferase Reporter Assay in RGK1-Transfected ARE Stable Clones with Transient Transfection by MnSOD and Vector cDNA

**Figure 7:**
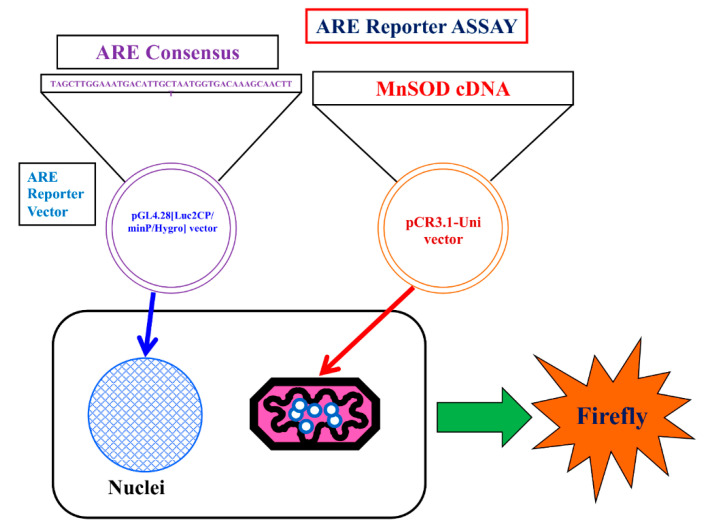
Schema of the Luciferase reporter assay for ARE. ARE consensus was stably transfected in the cells. hMnSOD vector is transiently transfected in the cells. Using this method, whether mtROS promote LAR II can be examined. In hMnSOD vector-transfected cells (MnSOD), if the fluorescence significantly decreased compared with that in vector-alone-transfected cells (Vector), these will suggest the amounts of mtROS became lower in MnSOD-transfected cells. As a results, ROS to bind keap1 will become less and the final amount of separated Keap1 to bind to ARE will became less. resulting less amount firefly luciferase activity (LAR II).

**Figure 8:**
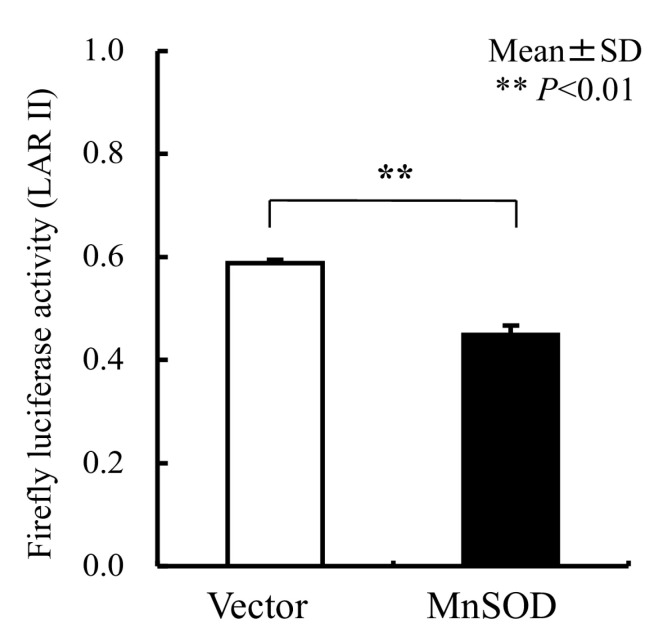
The results of the Luciferase reporter assay for ARE. In hMnSOD vector-transfected cells (MnSOD), the fluorescence significantly decreased compared with that in vector-alone-transfected cells (Vector), suggesting in MnSOD-transfected cells, the amounts of mtROS became lower, and, mtROS came out into cytosol from mitochondria and influenced the results of less ARE fluorescence signals. Bar: mean ± S.D.; *t*-test. **: *p* < 0.01.

**Figure 9:**
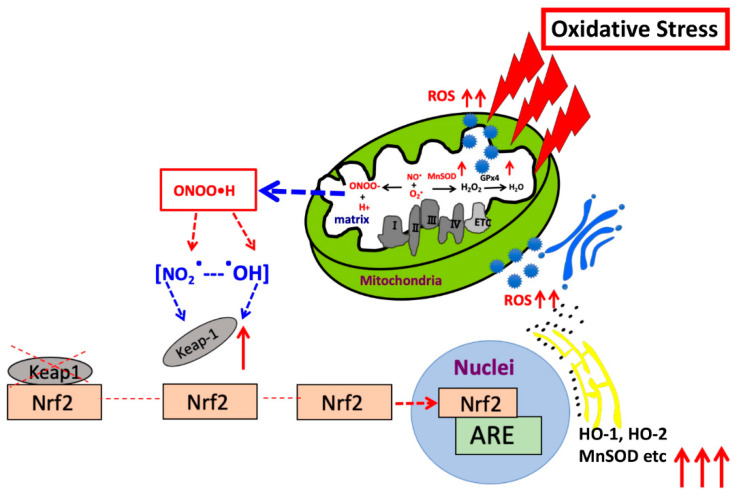
Schematic presentation indicating the mechanism by which mitochondrial ROS regulate Nrf2/Keap1 signaling in nucleus.

We used an ARE consensus-based dual luciferase reporter assay to determine the mitochondrial signaling pathway, which activates signals in nucleus outside mitochondria (Figure 7). It is noted that MnSOD locates inside mitochondria and scavenges ROS inside mitochondria. First, we constructed an ARE reporter vector and generated RGK1 cells that stably expressed this construct. Then, we transiently transfected those cells with hMnSOD or vector alone. Two days after the transfection, cell lysate was obtained, and the dual luciferase reporter assay was performed to measure firefly luciferase activity using LARII (Figure 7). Using this assay, if MnSOD transfection decrease the results of ARE luciferase intensity, then prove MnSOD lowered the signals in nucleus. The results showed that hMnSOD-transfected cells had significantly lower luciferase activity compared with cells transfected with vector alone (Figure 8), suggesting that ROS generated in mitochondria can move away from the mitochondrial membrane and activate cellular signals in the cytosol. The initiation of intracellular signaling by mitochondrial ROS can be proven only by MnSOD transfection: MnSOD reduces ROS inside mitochondria (mtROS), and if mtROS activate signals in cytosol or nucleus, the signal must be reduced by the increase in MnSOD amounts. A schematic of Nrf2/Keap1 signal transduction initiated by mitochondrial ROS is shown in Figure 9.

## 4. Discussion

In this paper, we could confirm that hMnSOD lowered the expression of Nrf2, Keap1, HO-1 and 2, MnSOD, GCL, GST, NQO1, probably through Nrf2-Keap1 activation, suggesting mitochondrial ROS regulates the signaling outside mitochondria, in nucleus.

Mitochondria are the major source of intracellular ROS [1,35]. Our group was the first to show that mitochondrial ROS (mtROS) cause apoptosis [20]. Murphy and Hartley previously revealed that mitochondrial dysfunction contributes to the pathology of many common disorders, including neurodegeneration, metabolic disease, heart failure, ischemia–reperfusion injury, and protozoal infections [52].

We recently showed that that mitochondrial ROS can induce the expression of GATA proteins, given that MnSOD transfection, which causes a decrease in superoxide levels, resulted in a reduction in the expression of GATA1, GATA3, GATA4 and GATA5 [46] (Figure S1). It is known that NF-κB controls GATA3 [53]. In cells, signal transduction networks act to maintain homeostasis and prevent major changes in intracellular status including alterations to redox potentials. Among the multiple pathways involved, signal transduction via NF-κB appears to play a key role during inflammation, immunity, development, cell growth, and survival. NF-κB regulates over 100 genes, including those with both antioxidant and pro-oxidant functions [46]. Tumor necrosis factor-α (TNF-α) is a well-established inducer of NF-κB, and induction occurs in a ROS-dependent manner [54]. Although antioxidant TNF-α has been reported to induce NF-κB activation [55], there exists overwhelming evidence for a key role of numerous oxidants in this process, which is postulated to be clinically important in the manifestation of several diseases [56,57,58,59,60,61,62,63,64,65]. The protein NF-κB essential modulator (NEMO, known as an inhibitor of NF-κB kinase subunit gamma, IKK-γ), is a subunit of the IκB kinase complex that activates NF-κB when present in a dimeric (disulfide-bonded) form. The formation of these disulfide bonds involves Cys54 and Cys347, and the treatment of cells with hydrogen peroxide enhances the formation of NEMO dimers. These findings suggest that oxidants can activate the NF-κB-related systems [66]. In our study, lowered mtROS resulted lowered GATAs expression through c less activation by the mtROS. Therefore, lowered mtROS results in the lower activities of both Nrf2-Keap1 signaling axis and NF-κB signaling axis, both the major intracellular signaling pathways.

This study confirms that mtROS activate Nrf2/Keap1 signaling. A schematic figure outlining Nrf2/Keap1 signaling is shown in Figure S2, which indicates that ROS activates Keap1 signaling as a result of Keap1 oxidative modification, consequently Nrf2 unbinds from Keap1. Nrf2/Keap1 signaling has been hypothesized in detail by Kasai et al. [67]. Many researchers have hypothesized that superoxide generated in mitochondria diffuses out from the mitochondria and activates cellular signals [46]. This phenomenon can be demonstrated by changing the amounts of mitochondrial superoxide, via a change in MnSOD expression [46]. The findings of this study and our previous work [46] suggest that superoxide generated in mitochondria can activate transduction signals in cytosol (Figure 2, Figure 3, Figure 4, Figure 5, Figure 6 and Figure S1) and increase ROS concentration, which, in turn, passes one or two membranes and activates GATA, Nrf2/Keap1 and other proteins and pathways.

## 5. Conclusions

In RGK1 cells where ROS production is increased, intracellular signaling, such as, Nrf2, Keap1, HO-1 and 2, MnSOD, GCL, GST, NQO1, GATA1, GATA3, GATA4, and GATA5 increases. This augmentation of signaling can be suppressed by MnSOD transfection. The results suggest that mitochondrial ROS can move out from mitochondria into the cytosol, and regulate various intracellular signals, placing them in a central position in controlling cellular signaling (Figure 9).

## Figures and Tables

**Table 1:** Primer sequences for detection of human and rat MnSOD genes after hMnSOD gene transfection.

Human MnSOD	F	5′ TTCTGGACAAACCTCAGCCCTAACGGT 3′
	R	5′ AACAGATGCAGCCGTCAGCTTCTCCTTAAA 3′
Rat MnSOD (control)	F	5′ GATTGATGTGTGGGAGCACGCTTACTAT 3′
	R	5′ GCAAACTATGTATCTTTGGCTAACATTCTC 3′

## Data Availability

All data are shown in this paper.

## Supplementary Materials

The following supporting information can be downloaded at: https://www.mdpi.com/article/10.3390/biom13030445/s1, Figure S1: Results of immunocytochemical staining with anti-GATA1, -GATA3, -GATA4, and -GATA5 antibodies. The fluorescence intensity for GATA1, GATA3, GATA4, and GATA5 is significantly lower in MnSOD-transfected cells than control cells. Bar: mean ± S.E.; *t*-test. **: *p* < 0.001. Reproduced from [46] with permission. Figure S2: Schematic of Nrf2/Keap1 signaling. ROS activate Keap1 signaling by Keap1 oxidative modification, and as a result, Nrf2 separates from Keap1.

## References

[1] Majima H., Indo H., Suenaga S., Kaneko T., Matsui H., Yen H.-C., Ozawa T., Naito Y., Suematsu M., Yoshikawa T. (2010). Mitochondria as source of free radicals. Free Radical Biology in Digestive Diseases.

[2] Majima H.J., Indo H.P., Nakanishi I., Suenaga S., Matsumoto K., Matsui H., Minamiyama Y., Ichikawa H., Yen H.C., Hawkins C.L. (2016). Chasing great paths of Helmut Sies “Oxidative Stress”. Arch. Biochem. Biophys..

[3] Warburg O. (1956). On the origin of cancer cells. Science.

[4] Vaupel P., Multhoff G. (2021). Revisiting the Warburg effect: Historical dogma versus current understanding. J. Physiol..

[5] Indo H.P., Davidson M., Yen H.-C., Suenaga S., Tomita K., Nishii T., Higuchi M., Koga Y., Ozawa T., Majima H.J. (2007). Evidence of ROS generation by mitochondria in cells with impaired electron transport chain and mitochondrial DNA damage. Mitochondrion.

[6] DiMauro S., Schon E.A. (2003). Mitochondrial respiratory-chain diseases. N. Engl. J. Med..

[7] Salway J.G. (2017). 6. Metbolism of one molecule of glucose yields 31 (or should it be 38?) molecules of ATP. Metabolism at a Glance.

[8] Boveris A., Chance B. (1973). The mitochondrial generation of hydrogen peroxide. General properties and effect of hyperbaric oxygen. Biochem. J..

[9] Takeshige K., Minakami S. (1979). NADH- and NADPH-dependent formation of superoxide anions by bovine heart submitochondrial particles and NADH-ubiquinone reductase preparation. Biochem. J..

[10] Beyer R.E. (1992). An analysis of the role of coenzyme Q in free radical generation and as an antioxidant. Biochem. Cell Biol..

[11] Wallace D.C. (1997). Mitochondrial DNA in aging and disease. Sci. Am..

[12] Grivennikova V.G., Vinogradov A.D. (2006). Generation of superoxide by the mitochondrial Complex I. Biochim. Biophys. Acta.

[13] Muller F.L., Liu Y., Van Remmen H. (2004). Complex III releases superoxide to both sides of the inner mitochondrial membrane. J. Biol. Chem..

[14] James A.M., Smith R.A., Murphy M.P. (2004). Antioxidant and prooxidant properties of mitochondrial Coenzyme Q. Arch. Biochem. Biophys..

[15] Yen H.C., Li S.H., Majima H.J., Huang Y.H., Chen C.P., Liu C.C., Tu Y.C., Chen C.W. (2011). Up-regulation of antioxidant enzymes and coenzyme Q_10_ in a human oral cancer cell line with acquired bleomycin resistance. Free Radic. Res..

[16] Linnane A.W., Kios M., Vitetta L. (2007). Coenzyme Q_10_—Its role as a prooxidant in the formation of superoxide anion/hydrogen peroxide and the regulation of the metabolome. Mitochondrion.

[17] Park I.H., Lee K.S., Ro J. (2015). Effects of second and subsequent lines of chemotherapy for metastatic breast cancer. Clin. Breast Cancer.

[18] Hosoi Y., Sakamoto K. (1993). Suppressive effect of low dose total body irradiation on lung metastasis: Dose dependency and effective period. Radiother. Oncol..

[19] Feng L., Qin L., Guo D., Deng D., Lu F., Li H., Bao N., Yang X., Ding H., Li J. (2017). Immunological mechanism of low-dose priming radiation resistance in walker-256 tumor model mice. Exp. Ther. Med..

[20] Majima H.J., Oberley T.D., Furukawa K., Mattson M.P., Yen H.C., Szweda L.I., St Clair D.K. (1998). Prevention of mitochondrial injury by manganese superoxide dismutase reveals a primary mechanism for alkaline-induced cell death. J. Biol. Chem..

[21] St Clair D.K., Oberley T.D., Ho Y.S. (1991). Overproduction of human Mn-superoxide dismutase modulates paraquat-mediated toxicity in mammalian cells. FEBS Lett..

[22] Wong G.H., Elwell J.H., Oberley L.W., Goeddel D.V. (1989). Manganous superoxide dismutase is essential for cellular resistance to cytotoxicity of tumor necrosis factor. Cell.

[23] Hirose K., Longo D.L., Oppenheim J.J., Matsushima K. (1993). Overexpression of mitochondrial manganese superoxide dismutase promotes the survival of tumor cells exposed to interleukin-1, tumor necrosis factor, selected anticancer drugs, and ionizing radiation. FASEB J..

[24] Sun J., Chen Y., Li M., Ge Z. (1998). Role of antioxidant enzymes on ionizing radiation resistance. Free Radic. Biol. Med..

[25] Leach J.K., Van Tuyle G., Lin P.S., Schmidt-Ullrich R., Mikkelsen R.B. (2001). Ionizing radiation-induced, mitochondria-dependent generation of reactive oxygen/nitrogen. Cancer Res..

[26] Motoori S., Majima H.J., Ebara M., Kato H., Hirai F., Kakinuma S., Yamaguchi C., Ozawa T., Nagano T., Tsujii H. (2001). Overexpression of mitochondrial manganese superoxide dismutase protects against radiation-induced cell death in the human hepatocellular carcinoma cell line HLE. Cancer Res..

[27] Epperly M.W., Gretton J.E., Sikora C.A., Jefferson M., Bernarding M., Nie S., Greenberger J.S. (2003). Mitochondrial localization of superoxide dismutase is required for decreasing radiation-induced cellular damage. Radiat. Res..

[28] Indo H.P., Inanami O., Koumura T., Suenaga S., Yen H.C., Kakinuma S., Matsumoto K., Nakanishi I., St Clair W., St Clair D.K. (2012). Roles of mitochondria-generated reactive oxygen species on X-ray-induced apoptosis in a human hepatocellular carcinoma cell line, HLE. Free Radic. Res..

[29] Hirai F., Motoori S., Kakinuma S., Tomita K., Indo H.P., Kato H., Yamaguchi T., Yen H.C., St Clair D.K., Nagano T. (2004). Mitochondrial signal lacking manganese superoxide dismutase failed to prevent cell death by reoxygenation following hypoxia in a human pancreatic cancer cell line, KP4. Antioxid. Redox Signal..

[30] St Clair D.K., Jordan J.A., Wan X.S., Gairola C.G. (1994). Protective role of manganese superoxide dismutase against cigarette smoke-induced cytotoxicity. J. Toxicol. Environ. Health.

[31] St Clair D.K., Wan X.S., Oberley T.D., Muse K.E., St Clair W.H. (1992). Suppression of radiation-induced neoplastic transformation by overexpression of mitochondrial superoxide dismutase. Mol. Carcinog..

[32] Wispé J.R., Warner B.B., Clark J.C., Dey C.R., Neuman J., Glasser S.W., Crapo J.D., Chang L.Y., Whitsett J.A. (1992). Human Mn-superoxide dismutase in pulmonary epithelial cells of transgenic mice confers protection from oxygen injury. J. Biol. Chem..

[33] Yen H.C., Oberley T.D., Vichitbandha S., Ho Y.S., St Clair D.K. (1996). The protective role of manganese superoxide dismutase against adriamycin-induced acute cardiac toxicity in transgenic mice. J. Clin. Investig..

[34] Majima H.J., Indo H.P., Tomita K., Iwashita Y., Suzuki H., Masuda D., Shimazu T., Tanigaki F., Umemura S., Yano S. (2009). Bio-assessment of risk in long-term manned space exploration—Cell death factors in space radiation and/or microgravity: A review. Bio. Sci. Space.

[35] Indo H.P., Yen H.C., Nakanishi I., Matsumoto K., Tamura M., Nagano Y., Matsui H., Gusev O., Cornette R., Okuda T. (2015). A mitochondrial superoxide theory for oxidative stress diseases and aging. J. Clin. Biochem. Nutr..

[36] Zhang D.X., Gutterman D.D. (2007). Mitochondrial reactive oxygen species-mediated signaling in endothelial cells. Am. J. Physiol. Heart Circ. Physiol..

[37] Finkel T. (2012). Signal transduction by mitochondrial oxidants. J. Biol. Chem..

[38] Cosentino-Gomes D., Rocco-Machado N., Meyer-Fernandes J.R. (2012). Cell signaling through protein kinase C oxidation and activation. Int. J. Mol. Sci..

[39] Holmström K.M., Finkel T. (2014). Cellular mechanisms and physiological consequences of redoxdependent signalling. Nat. Rev. Mol. Cell Biol..

[40] Chandel N.S. (2014). Mitochondria as signaling organelles. BMC Biol..

[41] Chandel N.S. (2015). Evolution of mitochondria as signaling organelles. Cell Metab..

[42] Shadel G.S., Horvath T.L. (2015). Mitochondrial ROS signaling in organismal homeostasis. Cell.

[43] Itoh K., Ye P., Matsumiya T., Tanji K., Ozaki T. (2015). Emerging functional cross-talk between the Keap1-Nrf2 system and mitochondria. J. Clin. Biochem. Nutr..

[44] Kobayashi I., Kawano S., Tsuji S., Matsui H., Nakama A., Sawaoka H., Masuda E., Takei Y., Nagano K., Fusamoto H. (1996). RGM1, a cell line derived from normal gastric mucosa of rat. In Vitro Cell. Dev. Biol. Anim..

[45] Shimokawa O., Matsui H., Nagano Y., Kaneko T., Shibahara T., Nakahara A., Hyodo I., Yanaka A., Majima H.J., Nakamura Y. (2008). Neoplastic transformation and induction of H^+^,K^+^-adenosine triphosphatase by *N*-methyl-*N*′-nitro-*N*-nitrosoguanidine in the gastric epithelial RGM-1 cell line. In Vitro Cell. Dev. Biol. Anim..

[46] Indo H.P., Hawkins C.L., Nakanishi I., Matsumoto K.I., Matsui H., Suenaga S., Davies M.J., St Clair D.K., Ozawa T., Majima H.J. (2017). Role of mitochondrial reactive oxygen species in the activation of cellular signals, molecules, and function. Handb. Exp. Pharmacol..

[47] Setsukinai K., Urano Y., Kakinuma K., Majima H.J., Nagano T. (2003). Development of novel fluorescence probes that can reliably detect reactive oxygen species and distinguish specific species. J. Biol. Chem..

[48] Beauchamp C., Fridovich I. (1971). Superoxide dismutase: Improved assays and an assay applicable to acrylamide gels. Anal. Biochem..

[49] Wasserman W.W., Fahl W.E. (1997). Functional antioxidant responsive elements. Proc. Natl. Acad. Sci. USA.

[50] Majima H.J., Indo H.P., Suenaga S., Matsui H., Yen H.C., Ozawa T. (2011). Mitochondria as possible pharmaceutical targets for the effects of vitamin E and its homologues in oxidative stress-related diseases. Curr. Pharm. Des..

[51] Li X., Deng A., Liu J., Hou W. (2018). The role of Keap1-Nrf2-ARE signal pathway in diabetic retinopathy oxidative stress and related mechanisms. Int. J. Clin. Exp. Pathol..

[52] Murphy M.P., Hartley R.C. (2018). Mitochondria as a therapeutic target for common pathologies. Nat. Rev. Drug Discov..

[53] Das J., Chen C.H., Yang L., Cohn L., Ray P., Ray A. (2001). A critical role for NF-κB in GATA3 expression and TH2 differentiation in allergic airway inflammation. Nat. Immunol..

[54] Morgan M.J., Liu Z.G. (2011). Crosstalk of reactive oxygen species and NF-κB signaling. Cell Res..

[55] Jamaluddin M., Wang S., Boldogh I., Tian B., Brasier A.R. (2007). TNF-α-induced NF-κB/RelA Ser^276^ phosphorylation and enhanceosome formation is mediated by an ROS-dependent PKAc pathway. Cell. Signal..

[56] Liu J., Yoshida Y., Yamashita U. (2008). DNA-binding activity of NF-κB and phosphorylation of p65 are induced by *N*-acetylcysteine through phosphatidylinositol (PI) 3-kinase. Mol. Immunol..

[57] Schieven G.L., Kirihara J.M., Myers D.E., Ledbetter J.A., Uckun F.M. (1993). Reactive oxygen intermediates activate NF-κB in a tyrosine kinase-dependent mechanism and in combination with vanadate activate the p56lck and p59fyn tyrosine kinases in human lymphocytes. Blood.

[58] Peng H.B., Libby P., Liao J.K. (1995). Induction and stabilization of IκBα by nitric oxide mediates inhibition of NF-κB. J. Biol. Chem..

[59] Li N., Karin M. (1999). Is NF-κB the sensor of oxidative stress?. FASEB J..

[60] Bowie A., O'Neill L.A. (2000). Oxidative stress and nuclear factor-κB activation: A reassessment of the evidence in the light of recent discoveries. Biochem. Pharmacol..

[61] Kamata H., Manabe T., Oka S., Kamata K., Hirata H. (2002). Hydrogen peroxide activates IκB kinases through phosphorylation of serine residues in the activation loops. FEBS Lett..

[62] Schoonbroodt S., Piette J. (2000). Oxidative stress interference with the nuclear factor-κB activation pathways. Biochem. Pharmacol..

[63] Takada Y., Mukhopadhyay A., Kundu G.C., Mahabeleshwar G.H., Singh S., Aggarwal B.B. (2003). Hydrogen peroxide activates NF-κB through tyrosine phosphorylation of IκBα and serine phosphorylation of p65: Evidence for the involvement of IκBα kinase and Syk protein-tyrosine kinase. J. Biol. Chem..

[64] Gloire G., Legrand-Poels S., Piette J. (2006). NF-κB activation by reactive oxygen species: Fifteen years later. Biochem. Pharmacol..

[65] Gloire G., Piette J. (2009). Redox regulation of nuclear post-translational modifications during NF-κB activation. Antioxid. Redox Signal..

[66] Herscovitch M., Comb W., Ennis T., Coleman K., Yong S., Armstead B., Kalaitzidis D., Chandani S., Gilmore T.D. (2008). Intermolecular disulfide bond formation in the NEMO dimer requires Cys54 and Cys347. Biochem. Biophys. Res. Commun..

[67] Kasai S., Shimizu S., Tatara Y., Mimura J., Itoh K. (2020). Regulation of Nrf2 by mitochondrial reactive oxygen species in physiology and pathology. Biomolecules.

