# Supplementary material for: Evidence of Nrf2/Keap1 Signaling Regulation by Mitochodria-Generated Reactive Oxygen Species in RGK1 Cells
Source: Biomolecules. 2023 Feb 27;13(3):445. doi: 10.3390/biom13030445 (PMC10046053; doi:10.3390/biom13030445)
Supplement: Supplementary file 1 [file biomolecules-13-00445-s001.zip › biomolecules-2192038-supplementary.pdf]

Supplement materials

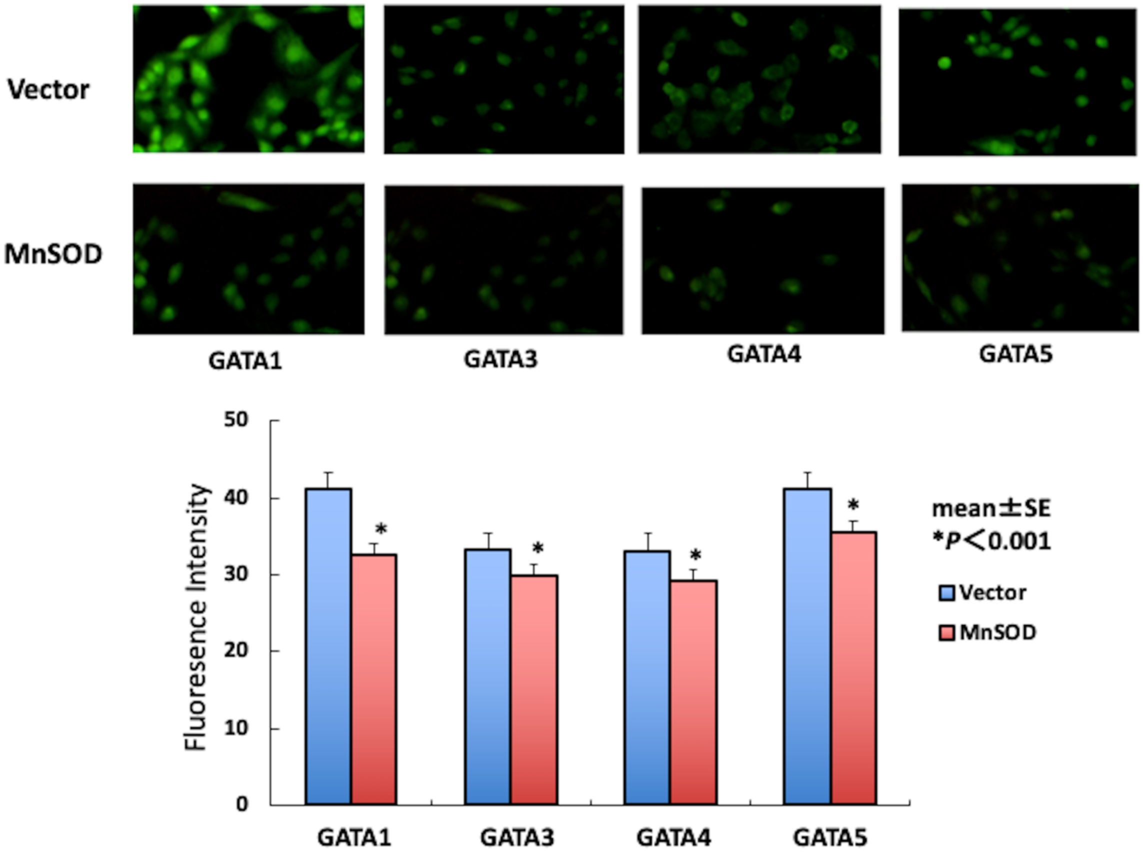

Figure S1. Results of Immuno-cyto-chemistry Staining detected by GATA1, 3, 4 ,and 5 antibodies. The fluorescence intensity for both HO-1 and HO-2 is significantly greater in RGK1 cells compared with that of RGM1 cells. *Bar*: mean ± S.E.; t-test. **: $p < 0.001$. Remounted from Ref. 46 with permission.

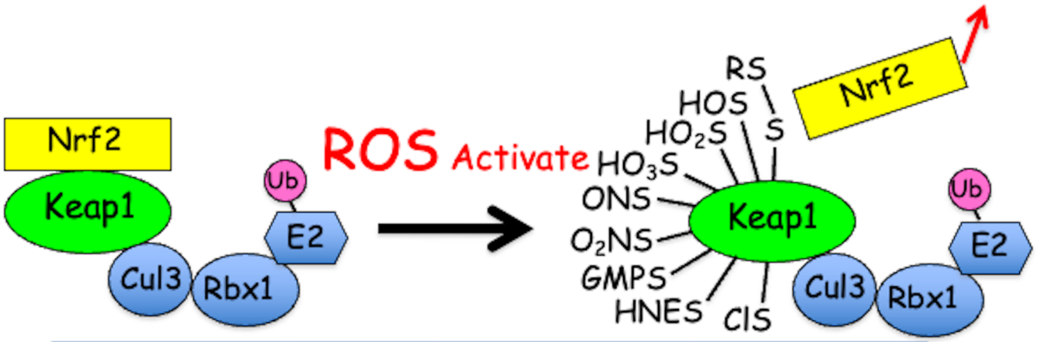

**Disulfides**	**-S–SR**	**Nitration**	**-SNO_2**
**Sulfenic acid**	**-SOH**	**S-guanylation**	**-SGMP**
**Sulfinic acid**	**-SO_2H**	**HNE modification**	**-SHNE**
**Sulfonic acid**	**-SO_3H**	**Sulfenyl halides**	**-SX (X = Cl etc.)**
**Nitroso adducts**	**-SNO**		

Figure S2. Schematic figure of fNrf2-keap1 signaling. ROS activate keap1 signaling by keap1 oxidative modification as a results Nrf2 separates from keap1.
